# 3-Year outcomes in patients with heavily calcified lesions undergoing percutaneous coronary intervention using cutting balloons

**DOI:** 10.1186/s12872-022-02622-9

**Published:** 2022-04-21

**Authors:** Wei Liu, Yutong Yao, Zhi Jiang, Longhai Tian, Bo Song, Hui Liu, Shiyan Deng, Rui Luo, Fang Wei

**Affiliations:** 1 Cardiology Department, Guizhou Provincial People’s Hospital, No. 83 East Zhongshan Road, Yunyan District, Guiyang, 550002 China; 2 Guizhou Provincial Cardiovascular Disease Institute, Guiyang, China

**Keywords:** Calcified lesion, Coronary artery disease, Percutaneous coronary intervention, Modified balloon

## Abstract

**Background:**

Percutaneous coronary intervention (PCI) of heavily calcified lesions (HCLs) is associated with higher complication rates and worse clinical outcomes. Cutting balloon (CB) has been widely used for HCLs, but patients’ prognosis had not been fully clarified. The study aimed to compare 3-year clinical outcomes between patients with HCLs that are treated with CBs and those with non-HCLs.

**Method:**

Patients who underwent PCI in Guizhou Provincial People’s Hospital from June 2015 to September 2018 were retrospectively included. HCL was defined as radiopaque and high-pressure undilatable lesions. CBs were routinely used in combination with non-compliant balloons for the HCLs. Major adverse cardiac event (MACE) and target vessel failure (TVF) were assessed at 3-year follow-up.

**Result:**

Among 2432 patients included in the study, 210(8.6%) had HCLs with a procedural success rate of 91.0%. The patients with HCLs had a higher incidence of MACE (23.3% vs. 10.8%, *P* < 0.001) than those with non-HCLs. By propensity score matching, 172 patients with HCLs were 1:1 paired to those with non-HCLs, and their PCI vessels were exactly matched. The MACE and TVF were significantly higher in the patients with HCLs than those with non-HCLs (MACE: 21.5% vs. 13.4%, *P* = 0.036; TVF: 19.8% vs. 9.9%, *P* = 0.008). In the Cox regression analysis, HCL is independently associated with higher risks of MACE [HR: 1.72(1.01–2.94), *P* = 0.047], TVF [HR: 2.10(1.15–3.81), *P* = 0.015] and repeat revascularization [HR: 2.20(1.07–4.52), *P* = 0.032].

**Conclusion:**

Patients with HCLs undergoing PCI using CBs in combination with non-compliant balloons had higher risks of complications, procedural failure, and worse clinical outcomes at 3 years than those with non-HCLs.

**Supplementary Information:**

The online version contains supplementary material available at 10.1186/s12872-022-02622-9.

## Introduction

Severe coronary artery calcification is a major challenge associated with higher complication rates and worse clinical outcomes in patients undergoing percutaneous coronary intervention (PCI) [1]. In the past decades, technologies have been developed for severely calcified lesions, but there is scarce evidence that one device is superior to the others in improving clinical outcomes. Compared to atherectomy and lithotripsy, the cutting balloon (CB) is widely available, less expensive, and simpler to use [2]. It has 3 to 4 microblades attached to the surface longitudinally, improving vessel compliance by cracking the calcified plaques. In the recent study, the CBs were more effective in plaque modification than the scoring balloons [3]. The PREPARE-CALC (Comparison of Strategies to Prepare Severely Calcified Coronary Lesions) trial reported that the acute lumen gain was not significantly different between the rotational atherectomy (RA) and modified balloons [4]. CB is likely to be an underused and potentially useful adjunct in calcium modification [1]. However, the outcomes in the patients with calcified lesions that were treated by cutting balloons were less studied [5–7]. It is important to investigate the unique prognostic impact of CBs on calcified lesions.

The severity of calcified lesions was assessed by angiographic radiopacity and mobility [6–9]. However, radiopacity does not reveal the calcific arc angle or thickness, which could generate heterogeneity in the outcome of calcified lesions [10]. In the present study, heavily calcified lesion (HCL) is defined as radiopaque and high-pressure undilatable lesions. We aimed to compare the 3-year clinical outcomes between the patients with the HCLs that were treated by CBs and those with non-HCLs.

## Method

### Study population

A retrospective cohort of patients undergoing PCI in Guizhou Provincial People’s Hospital from June 2015 to September 2018 was included, with the permission of the institutional ethics committee. Patients who were older than 84 years, had EF less than 35%, refractory heart failure, cardiac shock, severe valvular dysfunction, COPD stage greater than 2, or hemodialysis were excluded, because they had a high risk of mortality in addition to the coronary lesions and PCI devices. Patients with lesions in bypass grafts or in-stent restenosis were excluded as the target lesions were not de novo coronary artery atherosclerosis. Patients with no stent or bare-metal stents implantation were excluded due to their concomitant disease and higher restenosis rate compared with the second-generation drug-eluting stents (DES). Patients with HCLs who were initially treated by rotational ablation were excluded because they did not meet the study aim.

### PCI procedure

All PCI procedures were performed following the ACC/AHA guidelines of coronary revascularization [11, 12]. Patients received dual antiplatelet therapy (DAPT) before and after PCI. Heparin 100u/kg body weight was administrated to maintain an ACT > 250 s or 200–250 s if glycoprotein (GP) IIb/IIIa antagonist was infused. HCL was defined as the radiopacities within the vascular wall at the site of the stenosis which was undilatable at 18 atm. by non-compliant balloons. When the HCLs were identified, the CBs (Flextome, Boston Scientific, MA) with diameter 0.25–0.5 mm less than the reference vessel were routinely utilized for plaque modification. CB dilation was performed by increasing 1 atm. every 5 s. If CBs failed to be delivered to the HCLs, dilation at higher pressure (> 18 atm.) by the non-compliant balloons was performed to allow further plaque modification by the CBs. Stenting was performed using the second-generation DES. The use of intravascular imaging devices and post-dilation were left to the discretion of the operators. Procedural failure was defined as the failure of lesion preparation or the presence of > 20% in-stent residual stenosis. The patients who had procedural failure were managed with RA, coronary artery bypass grafting, or optimized medication therapy.

### Patient follow-up

All patients were evaluated by clinic visit or by telephone interview at 30 days postoperatively, every 3 months for the first year, and every 6 months thereafter. Patients were advised to return for coronary angiography if clinical symptoms or documentations indicated myocardial ischemia.

### Propensity score matching

Propensity score matching (PSM) was applied to compare the clinical outcomes between patients with and without HCLs using R software (version 4.0.5, The R Foundation, Vienna, Austria) to conduct the logistic regression model (stats package, version 4.0.5). The patients with HCLs were 1:1 matched to those with non-HCLs using the nearest neighbor method by the caliper of 0.03, and the PCI vessels were exactly matched (MatchIt package, version 4.2.0). The most complex HCL was first designated as the target lesion in each patient with HCLs. After PSM, the non-HCL in the paired patient with non-HCL was designated as the control lesion. Then the target and control vessels were designated according to the lesions.

The balance test for PSM included standardized mean difference (SMD) (cobalt package, version 4.3.1), prognostic score, and c-statistic [13]. The variables with SMD less than 0.1 are considered to be between-group balanced. The prognostic score represents the baseline probability of adverse events in the absence of HCLs [14]. The SMD of the prognostic score less than 0.1 indicates that the baseline risk was between-group balanced. The c-statistic measured the discrimination of the logistic regression model that predicts HCLs. The area under the curve of receiver operating character (AUC-ROC) was calculated (pROC package, version 1.17.0). The value closer to 0.5 indicates that the confounding factors were more equally distributed between groups.

### Clinical outcomes

Patients oriented outcomes and device effectiveness were assessed in the study [15]. The former was the major adverse cardiac event (MACE), which was the composite occurrence of all-cause death, MI, or any repeat revascularization (ARR). The latter was the target vessel failure (TVF), which was the composite occurrence of cardiac death, MI, or target/control vessel revascularization (T/CVR). Due to the definition of target/control vessel, the TVF was only assessed in the PSM cohort. Death that could not be attributed to non-cardiac causes was considered as cardiac death. MI was defined according to the Fourth Universal Definition [16]. T/CVR was defined as any repeat PCI or surgical bypass of any segment of the target or control vessel. ARR was defined as any repeat PCI or surgical bypass of any segment of the vessels that had undergone PCI. Other outcomes included stent thrombosis (ST), stroke, and bleeding. ST was defined according to the consensus [15]. Any unexplained death or documented acute ischemia in the absence of any other obvious cause were considered ST. Bleeding was defined as types 3 and 5 according to the Bleeding Academic Research Consortium [17]. All outcomes were adjudicated by 3 independent cardiologists.

### Statistical analysis

Continuous variables were presented as mean ± standard deviation (SD) or median (quantile). The Student’s t-test was used for comparison if normally distributed; otherwise, the Mann–Whitney U test was used. Categorical variables were presented as frequency (percentage), and comparison was performed using the Chi-square test. A two-tailed *P* value of less than 0.05 was considered statistically significant. The time to event was estimated using the Kaplan–Meier analysis and the between-group differences were compared using the log-rank test [18]. The Cox regression analyses were conducted to adjust the hazard ratio of HCL. All statistical analysis was performed by the R software (version 4.0.5, The R Foundation, Vienna, Austria).

## Result

### Baseline and procedural characteristics

**Fig. 1:**
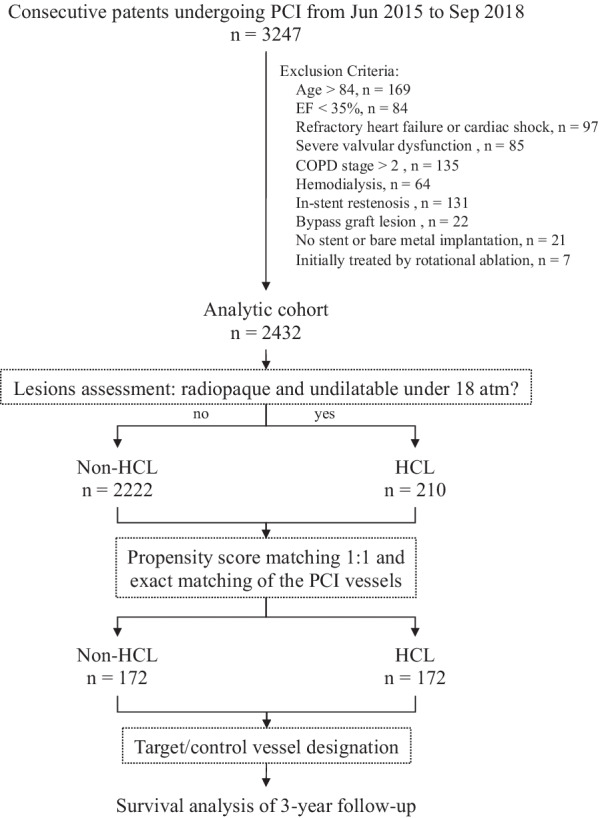
Patient flowchart. Abbreviations: COPD = chronic obstructive pulmonary disease; *EF*, ejection fraction, *HCL*, heavily calcified lesion; *PCI*, percutaneous coronary intervention

**Fig. 2:**
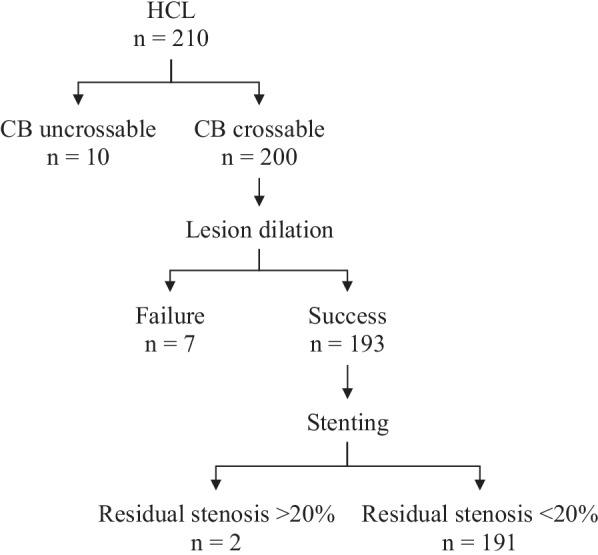
Effectiveness of CB in treating HCLs. Abbreviations: *CB*, cutting balloon; HCL, heavily calcified lesion

During the specified period, 3247 patients underwent PCI in our department (Fig. 1). 815 patients met the exclusion criteria, resulting in an eligible population of 2432 patients (male: 77.7%; age: 62.6 ± 10.8 years; HCL: 8.6%). The patients with HCLs had older age, worse renal function, and higher proportions of hypertension, diabetes, and dyslipidemia compared to those with non-HCLs (Table 1). The patients with HCLs had longer total lesion lengths, smaller minimum reference vessel diameters, higher proportions of multi-vessel PCI, higher incidence of procedural failure, and periprocedural complications (Table 2). 19(9.0%) patients with HCLs had procedural failure due to device uncrossable lesions (n = 10), undilatable lesions (n = 7), and the presence of > 20% in-stent residual stenosis (n = 2) (Fig. 2).

**Table 1:** Baseline characteristics

Baseline characteristics	Before PSM				After PSM			
	Non-HCL n = 2222	HCL n = 210	*P* value	SMD	Non-HCL n = 172	HCL n = 172	*P* value	SMD
Age, years	62.1 ± 10.8	67.1 ± 9.8	< 0.001	0.480	65.0 ± 11.5	66.0 ± 10.0	0.390	0.093
Body mass index, kg/m^2^	26.1 ± 4.8	25.49 ± 4.9	0.081	0.125	25.7 ± 4.8	25.7 ± 4.9	0.908	0.013
Male	1741 (78.4)	148 (70.5)	0.011	0.181	122 (70.9)	128 (74.4)	0.545	0.078
eGFR, ml/min/1.73m^2^	84.0 ± 26.5	73.6 ± 25.7	< 0.001	0.399	75.9 ± 24.0	77.2 ± 25.3	0.624	0.053
EF, %	54.1 ± 8.9	54.1 ± 9.2	0.930	0.006	53.8 ± 8.8	54.1 ± 9.2	0.745	0.035
Presentation with ACS	1740 (78.3)	171 (81.4)	0.334	0.078	143 (83.1)	139 (80.8)	0.674	0.061
Current smoker	799 (36.0)	69 (32.9)	0.411	0.065	52 (30.2)	58 (33.7)	0.563	0.075
Prior MI	395 (17.8)	46 (21.9)	0.164	0.104	34 (19.8)	37 (21.5)	0.790	0.043
Atrial fibrillation	173 (7.8)	23 (11.0)	0.139	0.109	11 (6.4)	14 (8.1)	0.678	0.067
Hypertension	1262 (56.8)	149 (71.0)	< 0.001	0.298	123 (71.5)	120 (69.8)	0.813	0.038
*Diabetes*	883 (39.7)	121 (57.6)	< 0.001	0.364	96 (55.8)	89 (51.7)	0.516	0.082
On insulin	366 (16.5)	51 (24.3)	0.006	0.195	43 (25.0)	35 (20.3)	0.367	0.111
Dyslipidemia	700 (31.5)	87 (41.4)	0.004	0.207	75 (43.6)	66 (38.4)	0.380	0.107

Values are mean ± SD or number (%)

*ACS*, acute coronary syndrome; *DAPT*, dual antiplatelet therapy; *EF*, ejection fraction; *eGFR*, estimated glomerular filtration rate; *HCL*, heavily calcified lesion; *MI*, myocardial infarction; *PSM*, propensity score matching; *RCA*, right coronary artery; *SMD*, standardized mean deviation; *ST*, stent thrombosis; *TLR*, target lesion revascularization

**Table 2:** Angiographic and PCI characteristics

Angiographic and PCI characteristics	Before PSM				After PSM			
	Non-HCL n = 2222	HCL n = 210	*P* value	SMD	Non-HCL n = 172	HCL n = 172	*P* value	SMD
Multi-vessel disease	542 (24.4)	85 (40.5)	< 0.001	0.349	54 (31.4)	54 (31.4)	1.000	< 0.001
*PCI vessels*								
LM	90 (4.1)	16 (7.6)	0.025	0.153	7 (4.1)	7 (4.1)	1.000	< 0.001
LM bifurcate	65 (2.9)	9 (4.3)	0.375	0.073	5 (2.9)	5 (2.9)	1.000	< 0.001
LAD	1418 (63.8)	139 (66.2)	0.542	0.050	108 (62.8)	108 (62.8)	1.000	< 0.001
LCX	560 (25.2)	70 (33.3)	0.013	0.179	52 (30.2)	52 (30.2)	1.000	< 0.001
RCA	844 (38.0)	95 (45.2)	0.047	0.148	69 (40.1)	69 (40.1)	1.000	< 0.001
Total lesion length, mm	33.3 ± 24.7	46.6 (28.6)	< 0.001	0.497	36.9 ± 25.1	39.5 ± 22.9	0.311	0.109
Minimum reference vessel diameter, mm	2.94 ± 0.41	2.86 ± 0.37	0.004	0.216	2.87 ± 0.40	2.88 ± 0.38	0.757	0.033
*Target/control vessel*^*a*^								
LM		12 (5.7)			6 (3.5)	6 (3.5)	1.000	< 0.001
LM bifurcate		9 (4.3)			5 (2.9)	5 (2.9)		
LAD		97 (46.2)			82 (47.7)	82 (47.7)	1.000	< 0.001
LCX		38 (18.1)			31 (18.0)	31 (18.0)	1.000	< 0.001
RCA		84 (40.0)			64 (37.2)	64 (37.2)	1.000	< 0.001
Complex lesion (type B2 and C)^a^		203 (96.7)			147 (85.5)	166 (96.5)	0.001	0.393
Target/control lesion length, mm^a^		31.3 ± 11.0			26.3 ± 12.6	30.1 ± 13.0	0.006	0.299
Minimum stent diameter, mm	2.94 (0.37)	2.85 (0.34)	0.001	0.265	2.88 (0.35)	2.86 (0.35)	0.540	0.068
Post-dilation	1628 (73.3)	188 (89.5)	< 0.001	0.427	128 (74.4)	153 (89.0)	0.001	0.383
IVUS	77 (3.5)	10 (4.8)	0.440	0.065	3 (1.7)	8 (4.7)	0.220	0.166
Procedural failure	18 (0.8)	19 (9.0)	< 0.001	0.388	2 (1.2)	13 (7.6)	0.008	0.317
*Periprocedural complications*	255 (11.5)	41 (19.5)	0.001	0.224	16 (9.3)	35 (20.3)	0.006	0.315
Coronary perforation	9 (0.4)	3 (1.4)	0.132	0.108	0 (0.0)	2 (1.2)	0.478	0.153
Tamponade	3 (0.1)	2 (1.0)	0.089	0.111	0 (0.0)	2 (1.2)	0.478	0.153
Coronary dissection	56 (2.5)	11 (5.2)	0.038	0.141	7 (4.1)	9 (5.2)	0.798	0.055
Slow/no flow	59 (2.7)	13 (6.2)	0.007	0.173	2 (1.2)	13 (7.6)	0.008	0.317
Periprocedural MI	205 (9.3)	32 (15.2)	0.007	0.184	9 (5.2)	26 (15.1)	0.004	0.331

Values are mean ± SD or number (%)

*HCL*, heavily calcified lesion; *IVUS*, intravascular ultrasound; *LAD*, left anterior descending artery; *LCX*, left circumflex artery; *LM*, left main artery; *PCI*, percutaneous intervention; *PSM*, propensity score matching; *RCA*, right coronary artery; *SMD*, standard mean deviation

^a^The control vessels were designated in the patients with non-HCLs after PSM according to the target vessel of their paired patients with HCLs. Therefore, the control vessels were not applicable before PSM

### PSM and balance test

Logistic multivariate analysis revealed the risk factors of HCL (Additional file 1: Table S1). 172(81.9%) patients with HCLs were 1:1 matched to those with non-HCLs. The characteristics that were associated with the clinical outcomes had SMD less than 0.1 (Table 1). The AUC-ROC decreased from 0.729 (95% CI 0.697–0.760) to 0.569 (95% CI 0.509–0.630) after PSM (Additional file 1: Figure S1). The SMDs of the prognostic scores reduced approximately 90% (Additional file 1: Table S2). The balance test indicated that the baseline risks were more equally distributed in the PSM cohort. As the PCI vessels were exactly matched, the multi-vessel PCI, PCI vessels, and target/control vessel were completely balanced. The HCLs had significantly longer lesion length (30.1 ± 13.0 mm vs. 26.3 ± 12.6, *P* = 0.006) and greater degree of complexity (type B2 and C: 96.5% vs. 85.5%, *P* = 0.001) than the non-HCLs (Table 2). The patients with HCLs had higher rates of procedural failure (7.6% vs. 1.2%, *P* = 0.008) and periprocedural complications (20.3% vs, 9.3%, *P* = 0.001) than those with non-HCLs.

### Clinical outcomes

**Fig. 3:**
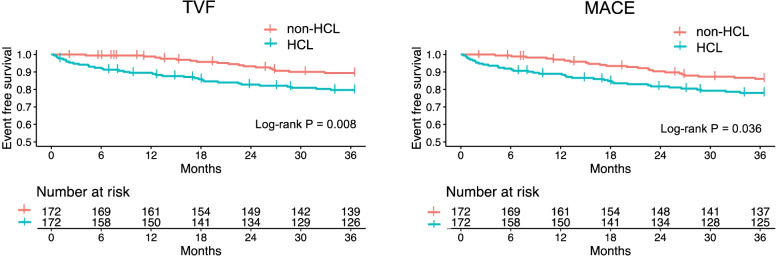
Kaplan–Meier survival analysis of 3-year TVF and MACE in the PSM cohort. Abbreviations: *HCL*, heavily calcified lesion; *MACE*, major adverse cardiac event; *PSM*, propensity score matching; *TVF*, target vessel failure

92.5% of the patients were followed up for 3 years. The clinical outcomes are presented in Table 3. In the total patients, MACE occurred in 239(10.8%) patients with non-HCLs and 49(23.3%) patients with HCLs. In the PSM cohort, the patients with HCLs had higher incidences of TVF (19.8% vs. 9.9%, *P* = 0.015), MACE (21.5% vs. 13.4%, *P* = 0.065), ARR (14.5% vs. 7.0%, *P* = 0.037) and T/CVR (12.8% vs. 5.2%, *P* = 0.024) than those with non-HCLs. The higher T/CVR in patients with HCLs was primarily caused by procedural failure. The Kaplan–Meier curves revealed worse outcomes of TVF (*P* = 0.008) and MACE (*P* = 0.036) in the patients with HCLs (Fig. 3). Bleeding and stroke events were similar between the groups. The incidence of ST was significantly higher in the patients with HCLs than those with non-HCLs in the total patients (3.3% vs. 0.8%, *P* = 0.001), but the difference was not statistically significant in the PSM cohort (3.5% vs. 1.2%, *P* = 0.283), probably due to the reduced sample size.

**Table 3:** 3-Year clinical outcomes

3-Years clinical outcomes	Before PSM			3-Years clinical outcomes	After PSM		
	Non-HCL n = 2222	HCL n = 210	*P* value		Non-HCL n = 172	HCL n = 172	*P* value
				TVF	17 (9.9)	34 (19.8)	0.015
*MACE*	239 (10.8)	49 (23.3)	< 0.001	*MACE*	23 (13.4)	37 (21.5)	0.065
All-cause death	97 (4.4)	17 (8.1)	0.023	All-cause death	8 (4.7)	14 (8.1)	0.271
Cardiac death	51 (2.3)	12 (5.7)	0.006	Cardiac death	3 (1.7)	11 (6.4)	0.056
MI	115 (5.2)	23 (11.0)	0.001	MI	11 (6.4)	20 (11.6)	0.132
ARR	125 (5.6)	34 (16.2)	< 0.001	ARR	12 (7.0)	25 (14.5)	0.037
				T/CVR	8 (4.7)	22 (12.8)	0.013
ST	17 (0.8)	7 (3.3)	0.001	ST	2 (1.2)	6 (3.5)	0.283
Bleeding	35 (1.6)	4 (1.9)	0.939	Bleeding	2 (1.2)	3 (1.7)	1.000
Stroke	22 (1.0)	2 (1.0)	1.000	Stroke	2 (1.2)	1 (0.6)	1.000
Follow-up < 3 year	167 (7.5)	11 (5.2)	0.283	Follow-up < 3 year	12 (7.0)	10 (5.8)	0.826

Values are number (%)

*ARR*, any repeat revascularization; *HCL*, heavily calcified lesion; *MACE*, major adverse cardiac event; *MI*, myocardial infarction; *PSM*, propensity score matching; *ST*, stent thrombosis; *TVF*, target lesion failure; *T/CVR*, target or control vessel revascularization

The Cox regression analysis was conducted to adjust the hazard ratio (HR) of HCLs to further verify the results (Table 4). In the PSM cohort, the HCL was independently associated with higher risks of TVF (HR: 2.10, 95% CI 1.15–3.81, *P* = 0.015), MACE (HR: 1.72, 95% CI 1.01–2.94, *P* = 0.047), ARR (HR: 2.20, 95% CI 1.07–4.52, *P* = 0.032) and T/CVR (HR: 2.63, 95% CI 1.14–6.07, *P* = 0.024). In the total patients, the HCL was also associated with higher risks of MACE (HR: 1.52, 95% CI 1.10–2.11, *P* = 0.011), cardiac death (2.06, 95% CI 1.04–4.05, *P* = 0.037) and ARR (HR: 2.08, 95% CI 1.39–3.11, *P* < 0.001). The HR of HCL on cardiac death in the PSM cohort (3.20, 95% CI 0.86–12.00, *P* = 0.084) was not statistically significant, probably due to the reduced sample size.

**Table 4:** Cox regression analysis of the HCL on 3-year clinical outcomes

HCL vs. non-HCL	Before PSM		HCL vs. non-HCL	After PSM	
	HR (95% CI)	*P* value		HR (95% CI)	*P* value
			TVF	2.10 (1.15–3.81)	0.015
*MACE*	1.52 (1.10–2.11)	0.011	*MACE*	1.72 (1.01–2.94)	0.047
All-cause death	1.25 (0.73–2.16)	0.415	All–cause death	1.56 (0.64–3.80)	0.332
Cardiac death	2.06 (1.04–4.05)	0.037	Cardiac death	3.20 (0.86–12.00)	0.084
MI	1.33 (0.83–2.13)	0.243	MI	2.19 (1.00–4.78)	0.050
ARR	2.08 (1.39–3.11)	< 0.001	ARR	2.20 (1.07–4.52)	0.032
			T/CVR	2.29 (1.03–5.11)	0.042

The HR in the total cohort was adjusted by patient age, male, EF, eGFR, total lesion length, minimum reference vessel diameter, presentation of ACS, prior MI, current smoker, diabetes, hypertension, and dyslipidemia. To adjust the HR in the PSM cohort, the total lesion length was replaced by target/control lesion length, and multi-vessel disease was added

*ACS*, acute coronary syndrome; *ARR*, any repeat revascularization; *CI*, confident interval; *HCL*, heavily calcified lesion; *HR*, hazard ratio; *MACE*, patient adverse event; *MI*, myocardial infarction; *PSM*, propensity score matching; *ST*, stent thrombosis; *TVF*, target lesion failure; *T/CVR*, target or control vessel revascularization

## Discussion

### Major findings

In the current study, we investigated the clinical outcomes of the patients with HCLs treated by CBs and compared their prognosis to those with non-HCLs. The HCLs accounted for 8.6% of the study population. The CBs in combination with non-compliant balloons had an overall 91.0% of procedural success in treating the HCLs. The HCLs had longer lesion lengths and higher degrees of lesion complexity than the non-HCLs. The patients with HCLs had significantly higher rates of complication and procedural failure than those with non-HCLs. HCL was independently associated with higher risks of TVF and MACE at 3 years.

### Discordance

In previous studies, calcified lesions were stratified by radiopacity and mobility [6–9, 19]. However, the exact calcific extent and distribution that could affect stent expansion and worsen long-term outcomes in such balloon dilatable lesions are still less understood [20]. Our definition of HCL included an additional criterion that the lesions were undilatable under 18 atm. by non-compliant balloons [21]. The criterion could imply the extent of intimal calcification with a large calcific arc and great thickness, indicating the necessity of a more aggressive device for plaque modification and the negative impact on the prognosis [10, 22]. It is noticed that the intravascular ultrasound (IVUS) was employed in only 3.6% of the total patients. The calcified lesions that were dilatable under 18 atm. or angiographically invisible were both defined as non-HCLs. They could less inhibit stent expansion and have less impact on the clinical outcomes [10]. Our definition could be more practical to identify challenging calcified lesions independent of intravascular imaging and reduce heterogenicity in the patients with HCLs.

We exactly matched the PCI vessels in the PSM analysis, generating a well-balanced cohort with clearly defined target and control lesions. However, PSM does not guarantee complete balance for all variables. The HCLs were significantly longer in lesion length, which was associated with an increased risk of in-stent restenosis [23]. The confounding factors were adjusted using the Cox regression analysis. The result indicated that the HCLs were independently associated with higher risks of adverse events.

### Comparing CB to other devices

Calcium fracture in the HCLs was associated with greater acute lumen gain and better stent expansion [24]. The optical coherence tomography thresholds for predicting calcium fracture were a maximum calcific arc angle of 227°, a minimum thickness of 0.67 mm by RA, and 0.24 mm by plain balloon angioplasty. By mathematical calculation, CB generates 157,000 times more pressure than a plain balloon [25]. The mechanism conferred CB greater effectiveness for calcium fracture than plain or scoring balloons [3, 26]. It is the rationale for the routine use of CB for HCLs in our hospital. The crossability of CBs is restricted by their large profile. In our study, the CBs were delivered to over half of the HCLs only after high-pressure dilation (> 18 atm.) by the non-compliant balloons. The PREPARE-CAL trial also reported that the modified balloons were less successful than the RA due to the larger profile of the former.[4] However, RA is considered a risk factor of coronary perforation and slow/no flow due to mechanical abrasion and debris [27]. In our study, the coronary perforation and tamponade rates were 1.4% and 1.0%, which were both lower than 4% and 3% in the PREPARE-CAL trial. Orbital atherectomy could be superior to RA in safety and calcium debulking, but more evidence is needed. Intravascular lithotripsy is a promising technology that selectively fractures noncompliant calcium and spares soft tissue, but its crossing profile is currently larger than CB [19].

### Clinical implication

Similar algorithms have been proposed for calcified lesions by combining multiple devices and intravascular imaging [1]. Although atherectomy is superior in procedural success in comparison with other technologies, it has not shown a reduction in long-term ischemic events. Moreover, atherectomy is not widely available and operators require specific training [2]. In our study, the CBs in combination with non-compliant balloons had procedural success in 91.0% of the patients with HCLs, suggesting that CB could be a simple and cost-effective adjunct. The MACE-trial suggested that the CBs or plain balloons could improve the outcomes in the patients with moderate calcification compared with RA [8]. A contemporary randomized trial using the newer generation CB (Wolverine, Boston Scientific, MA) and the super high-pressure balloon (OPN, SIS Medical, Switzerland) is warranted.

## Limitations

Our study has the following limitations. Firstly, it is a single-center retrospective study. The results were subject to unknown confounding factors. Secondly, IVUS was infrequently employed. The lesion morphology was not assessed in the study. The outcomes of the patients could have been improved if IVUS was used more frequently [28]. The generalizability of the major findings could be limited to other centers that routinely use intravascular imaging for complex lesions. Thirdly, the sample size was small. 18.1% of the patients with HCLs were unpaired in the PSM analysis. It could generate bias. Furthermore, only 7 patients with HCLs were initially treated by RA during the specified period. We were unable to compare the prognostic effect between the devices due to the limited cases. Whether the findings could be extrapolated to other devices requires further study to warrant.

## Conclusion

Despite CBs in combination with non-compliant balloons were routinely used for plaque modification in patients with HCLs, they still had higher risks of complications, procedural failure, and adverse events at 3 years than those with non-HCLs.

## Supplementary Information

**Additional file 1:** Table S1, S2 and Figure S1.

**Additional file 2:** The minimal dataset.

## Data Availability

The minimal datasets generated during the current study are available in the Additional file 2.

## Abbreviations

ARR: Any repeat revascularization
AUC-ROC: Area under the curve of receiver operating character
CB: Cutting balloon
CI: Confidential interval
DAPT: Dual antiplatelet therapy
DES: Drug-eluting stent
HCL: Heavily calcified lesion
HR: Hazard ratio
IVUS: Intravascular ultrasound
MACE: Major adverse cardiac event
MI: Myocardial infarction
PCI: Percutaneous coronary intervention
PSM: Propensity score matching
RA: Rotational atherectomy
SD: Standard deviation
SMD: Standardized mean difference
ST: Stent thrombosis
TVF: Target vessel failure
T/CVR: Target or control vessel revascularization

## References

[1] Shah M, Najam O, Bhindi R, De Silva K (2021). Calcium modification techniques in complex percutaneous coronary intervention. Circ Cardiovasc Interv.

[2] Beohar N, Kaltenbach LA, Wojdyla D, Pineda AM, Rao SV, Stone GW (2020). Trends in usage and clinical outcomes of coronary atherectomy: a report from the national cardiovascular data registry CathPCI Registry. Circ Cardiovasc Interv.

[3] Matsukawa R, Kozai T, Tokutome M, Nakashima R, Nishimura R, Matsumoto S (2019). Plaque modification using a cutting balloon is more effective for stenting of heavily calcified lesion than other scoring balloons. Cardiovasc Interv Ther.

[4] Abdel-Wahab M, Toelg R, Byrne RA, Geist V, El-Mawardy M, Allali A (2018). High-speed rotational atherectomy versus modified balloons prior to drug-eluting stent implantation in severely calcified coronary lesions. Circ Cardiovasc Interv.

[5] Giustino G, Mastoris I, Baber U, Sartori S, Stone GW, Leon MB (2016). Correlates and impact of coronary artery calcifications in women undergoing percutaneous coronary intervention with drug-eluting stents. JACC Cardiovasc Interv.

[6] Copeland-Halperin RS, Baber U, Aquino M, Rajamanickam A, Roy S, Hasan C (2018). Prevalence, correlates, and impact of coronary calcification on adverse events following PCI with newer-generation DES: Findings from a large multiethnic registry. Catheter Cardiovasc Interv.

[7] Jia S, Li J, Zhang C, Liu Y, Yuan D, Xu N (2020). Long-term prognosis of moderate to severe coronary artery calcification in patients undergoing percutaneous coronary intervention. Circ J.

[8] Sharma SK, Bolduan RW, Patel MR, Martinsen BJ, Azemi T, Giugliano G (2019). Impact of calcification on percutaneous coronary intervention: MACE-Trial 1-year results. Catheter Cardiovasc Interv.

[9] Hong X-L, Li Y, Zhou B-Q, Fu G-S, Zhang W-B (2021). Comparison of rotational atherectomy and modified balloons prior to drug-eluting stent implantation for the treatment of heavily calcified coronary lesions. Medicine (Baltimore).

[10] Wang X, Matsumura M, Mintz GS, Lee T, Zhang W, Cao Y (2017). In vivo calcium detection by comparing optical coherence tomography, intravascular ultrasound, and angiography. JACC Cardiovasc Imaging.

[11] Patel MR, Calhoon JH, Dehmer GJ, Grantham JA, Maddox TM, Maron DJ (2017). ACC/AATS/AHA/ASE/ASNC/SCAI/SCCT/STS 2016 appropriate use criteria for coronary revascularization in patients with acute coronary syndromes. J Am Coll Cardiol.

[12] Fihn SD, Blankenship JC, Alexander KP, Bittl JA, Byrne JG, Fletcher BJ (2014). 2014 ACC/AHA/AATS/PCNA/SCAI/STS focused update of the guideline for the diagnosis and management of patients with stable ischemic heart disease. J Am Coll Cardiol.

[13] Zhang Z, Kim HJ, Lonjon G, Zhu Y (2019). Balance diagnostics after propensity score matching. Ann Transl Med.

[14] Stuart EA, Lee BK, Leacy FP (2013). Prognostic score-based balance measures for propensity score methods in comparative effectiveness research. J Clin Epidemiol.

[15] Cutlip DE, Windecker S, Mehran R, Boam A, Cohen DJ, van Es G-A (2007). Clinical end points in coronary stent trials: a case for standardized definitions. Circulation.

[16] Thygesen K, Alpert JS, Jaffe AS, Chaitman BR, Bax JJ, Morrow DA (2018). Fourth universal definition of myocardial infarction (2018). Eur Heart J.

[17] Mehran R, Rao SV, Bhatt DL, Gibson CM, Caixeta A, Eikelboom J (2011). Standardized bleeding definitions for cardiovascular clinical trials: a consensus report from the Bleeding Academic Research Consortium. Circulation.

[18] Austin PC (2014). The use of propensity score methods with survival or time-to-event outcomes: reporting measures of effect similar to those used in randomized experiments. Stat Med.

[19] Sorini Dini C, Nardi G, Ristalli F, Mattesini A, Hamiti B, Di Mario C (2019). Contemporary Approach to Heavily Calcified Coronary Lesions. Interv Cardiol.

[20] Sharma SK, Vengrenyuk Y, Kini AS (2017). IVUS, OCT, and coronary artery calcification: is there a bone of contention?∗. JACC Cardiovasc Imaging.

[21] Madhavan MV, Tarigopula M, Mintz GS, Maehara A, Stone GW, Généreux P (2014). Coronary artery calcification: pathogenesis and prognostic implications. J Am Coll Cardiol.

[22] Mintz GS, Popma JJ, Pichard AD, Kent KM, Satler LF, Chuang YC (1995). Patterns of calcification in coronary artery disease. A statistical analysis of intravascular ultrasound and coronary angiography in 1155 lesions. Circulation.

[23] Cassese S, Byrne RA, Tada T, Pinieck S, Joner M, Ibrahim T (2014). Incidence and predictors of restenosis after coronary stenting in 10 004 patients with surveillance angiography. Heart.

[24] Fujino A, Mintz GS, Lee T, Hoshino M, Usui E, Kanaji Y (2018). Predictors of calcium fracture derived from balloon angioplasty and its effect on stent expansion assessed by optical coherence tomography. JACC Cardiovasc Interv.

[25] Ito S, Suzuki T, Suzuki T (2003). Adjunctive use of cutting balloon after rotational atherectomy in a young adult with probable Kawasaki disease. J Invasive Cardiol.

[26] Tang Z, Bai J, Su S-P, Wang Y, Liu M-H, Bai Q-C (2014). Cutting-balloon angioplasty before drug-eluting stent implantation for the treatment of severely calcified coronary lesions. J Geriatr Cardiol.

[27] Sharma SK, Tomey MI, Teirstein PS, Kini AS, Reitman AB, Lee AC (2019). North American expert review of rotational atherectomy. Circ Cardiovasc Interv.

[28] Shin D-H, Hong S-J, Mintz GS, Kim J-S, Kim B-K, Ko Y-G (2016). Effects of intravascular ultrasound-guided versus angiography-guided new-generation drug-eluting stent implantation. JACC Cardiovasc Interv.

