# Supplementary material for: 3-Year outcomes in patients with heavily calcified lesions undergoing percutaneous coronary intervention using cutting balloons
Source: BMC Cardiovasc Disord. 2022 Apr 21;22:187. doi: 10.1186/s12872-022-02622-9 (PMC9027752; doi:10.1186/s12872-022-02622-9)
Supplement: Supplementary file 1 — Additional file 1: Table S1, S2 and Figure S1. [file 12872_2022_2622_MOESM1_ESM.docx]

Table S1. Risk factors of HCL.

| Risk factors of HCL | OR (95% CI) | P value |
| --- | --- | --- |
| Age, 1 year increase | 1.04 (1.02-1.05) | <0.001 |
| eGFR, 1 ml/min increase | 0.99 (0.98-0.99) | <0.001 |
| Total lesion length, 1 mm increase | 1.01 (1.01-1.02) | <0.001 |
| Hypertension | 1.88 (1.37-2.60) | <0.001 |
| Diabetes | 1.54 (1.12-2.10) | 0.008 |
| Dyslipidemia | 1.50 (1.11-2.02) | 0.008 |
| Abbreviation: CI = confident interval; eGFR = estimated glomerular filtration rate; HCL = heavily calcified lesion; OR = odd ratio. | | |

Table S2. Prognostic scores before and after PSM.

|  | Before PSM | | | |  | After PSM | | | |
| --- | --- | --- | --- | --- | --- | --- | --- | --- | --- |
|  | non-HCL n = 2222 | HCL n = 210 | P value | SMD |  | non-HCL n = 172 | HCL n = 172 | P value | SMD |
| Prognostic score (MACE)^a^ | -2.86 ± 1.45 | -2.09 ± 1.43 | <0.001 | 0.536 |  | -2.31 ± 1.29 | -2.38 ± 1.32 | 0.607 | 0.055 |
| Prognostic score (TVF)^a^ | -3.23 ± 1.49 | -2.44 ± 1.47 | <0.001 | 0.537 |  | -2.68 ± 1.34 | -2.73 ± 1.32 | 0.713 | 0.040 |
| Values are mean ± SD. ^a^ Prognostic scores are presented as log-odds, representing the baseline risk of MACE or TVF in absence of HCLs. Risk factors were patient age, sex, eGFR, EF, current smoker, prior MI, hypertension, diabetes, dyslipidemia, total lesion length, and minimum reference vessel diameter.  Abbreviation: HCL = heavily calcified lesion; MACE = major adverse cardiac event; PSM = propensity score matching; SMD = standard mean deviation; TVF = target lesion failure. | | | | | | | | | |

Figure S1. ROC curves of the logistic regression model that predicts HCL before and after PSM. The AUC-ROC was shown in each plot.
